# Predictors of Response to CDK4/6i Retrial After Prior CDK4/6i Failure in ER+ Metastatic Breast Cancer

**DOI:** 10.21203/rs.3.rs-4237867/v1

**Published:** 2024-05-02

**Authors:** Nicholas Mai, Carlos H dos Anjos, Pedram Razavi, Anton Safonov, Sujata Patil, Yuan Chen, Joshua Z Drago, Shanu Modi, Jacqueline F Bromberg, Chau T Dang, Dazhi Liu, Larry Norton, Mark Robson, Sarat Chandarlapaty, Komal Jhaveri

**Affiliations:** 1 Department of Medicine, Memorial Sloan Kettering Cancer Center, New York, New York.; 2 Oncology Service, Department of Medicine, Hospital Sirio-Libanes, Sao Paulo, SP, Brazil.; 3 Department of Quantitative Health Sciences, Cleveland Clinic Taussig Cancer Institute, Cleveland, Ohio.; 4 Department of Epidemiology and Biostatistics, Memorial Sloan Kettering Cancer Center, New York, New York

## Abstract

After disease progression on endocrine therapy (ET) plus a CDK4/6 inhibitor, there is no standardized sequence for subsequent treatment lines for estrogen receptor positive (ER+) metastatic breast cancer (MBC). CDK4/6i retrial as a treatment strategy is commonplace in modern clinical practice; however, the available prospective data investigating this strategy have had inconclusive results. To frame this data in a real-world context, we performed a retrospective analysis assessing the efficacy of CDK4/6is in 195 patients who had previous exposure to CDK4/6i in a prior treatment line at our institution. Among patients who had stopped a CDK4/6i due to toxicity, CDK4/6i retrial either immediately after with a different CDK4/6i or in a further treatment line with the same initial CDK4/6i was both safe and effective, with a median time to treatment failure (TTF) of 10.1 months (95%CI, 4.8–16.9). For patients whose disease progressed on a prior CDK4/6i, we demonstrated comparable median TTFs for patients rechallenged with the same CDK4/6i (4.3 months, 95%CI 3.2–5.5) and with a different CDK4/6i (4.7 months, 95%CI 3.7–6.0) when compared to the recent PACE, PALMIRA, and MAINTAIN trials. Exploratory genomic analysis suggested that the presence of mutations known to confer CDK4/6i resistance, such as *TP53* mutations, *CDK4* amplifications, and *RB1* or *FAT1* loss of function mutations may be molecular biomarkers predictive of CDK4/6i retrial failure.

## INTRODUCTION

Estrogen-receptor positive (ER+) HER2 negative breast cancer is the most common breast cancer subtype, accounting for almost 60–65% of all breast cancers.^1^ ER+ breast cancer has the tendency for both locoregional and distant recurrence decades after initial diagnosis and treatment, with almost 20–30% of patients developing metastatic breast cancer (MBC) in this time.^2^ First-line treatment for ER+ MBC is a combination of endocrine therapy (ET) and cyclin-dependent kinase 4/6 inhibitors (CDK4/6i), where data from both the initial clinical trials and follow-up meta-analyses have shown significant improvements in both progression-free survival (PFS) and overall survival (OS) when compared to ET alone.^3–5^ However, despite the significant improvements in outcomes in ER+ MBC with the addition of CDK4/6i, resistance to both ET and CDK4/6i occurs almost universally after enough time.^6,7^

After progression on ET + CDK4/6i, there are many treatment options available for patients, but each subsequent treatment line has progressively diminishing efficacy and tolerability, while many are reliant upon specific molecular markers for treatment eligibility.^8^ However, despite the variety of options, there is no standard, optimal treatment after first line ET + CDK4/6i. Similar to how anti-HER2 targeting therapies can be offered again to patients with HER2+ disease even after they progress through first line trastuzumab + pertuzumab,^9^ it is of similar interest whether patients may benefit with continuing ET + CDK4/6i after initial progression or returning to it in subsequent treatment lines. Especially with increasing evidence for and usage of CDK4/6i in the adjuvant setting, understanding the circumstances where retrial would be useful becomes even more relevant. A few prospective clinical trials trying to answer this question already have preliminary, though conflicting results. Both the PACE and PALMIRA trials saw no improvements in PFS when comparing palbociclib + ET to ET alone in patients with ER+ MBC that had previously progressed on an aromatase inhibitor (AI) + palbociclib.^10,11^ In contrast, the MAINTAIN trial, which compared ribociclib + ET to ET alone showed a significant PFS benefit for the ribociclib combination therapy arm.^12^

To further clarify the clinical utility of CDK4/6i retrial after progression on first line ET + CDK4/6i therapy in ER+ MBC and to complement the prospective studies mentioned above, we performed a retrospective clinical and genomic analysis on patients treated at Memorial Sloan Kettering Cancer Center (MSK) with at least two documented treatment lines containing CDK4/6i. Our cohorts included patients retreated with the same CDK4/6i and patients treated with a different CDK4/6i. Here we report real-world efficacy and toxicity data of this CDK4/6i retrial treatment strategy coupled with a descriptive genomic analysis of the patients in our study.

## RESULTS

### Patients Characteristics

**Figure 1:**
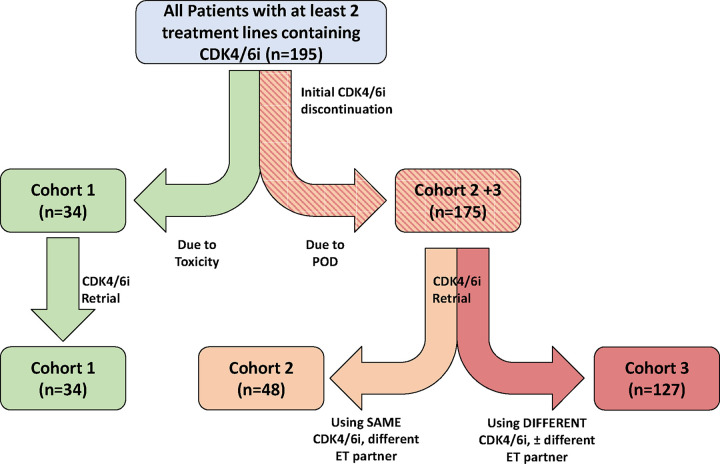
CDK4/6i Retrial Cohorts: A flow/CONSORT diagram outlining how patients were divided into cohorts for data analysis is shown here. From our 195 total patients, patients were first separated depending upon why their first CDK4/6i regimen was discontinued. Patients who discontinued therapy due to toxicity were considered Cohort 1. The remaining patients (who had stopped initial CDK4/6i due to progression of disease (POD)) were further separated depending upon what type of combination regimen was chosen on retrial. Cohort 2 represented patients who kept the same CDK4/6i but changed endocrine therapy (ET) partner. Cohort 3 represented patients who were treated with a different CDK4/6i. Of note, 14 patients were treated with 3 separate lines of therapy containing a CDK4/6i and therefore were documented as separate treatment instances (treatments 1 and 2 vs treatments 2 and 3). These individual patients ended up in multiple cohorts to account for their multiple treatment instances.

A total of 195 ER+/HER2− MBC patients treated at MSK with at least 2 separate treatment regimens containing a CDK4/6i between May 2014 and December 2020 were identified. Median age for all patients identified was 60. Patients were divided into three cohorts based upon the criteria outlined in Figure 1. Of the 195 total, 14 patients received three regimens containing a CDK4/6i and contributed to two different cohorts. Clinical characteristics for all patients in all cohorts are summarized in Table 1.

In Cohort 1 (n=34), the group of patients that had to stop first-line CDK4/6i purely due to toxicity, the most common toxicities leading to discontinuation were neutropenia (32%), skin rash (17.5%), and joint pain (17%). At the time of CDK4/6i retrial, 7 patients (20.5%) again had to stop treatment due to toxicity, with 6 out of 7 patients stopping for the same toxicity that caused discontinuation of first line treatment. 26.5% of patients had bone only disease and only 1 patient had brain metastasis at the time of CDK4/6i retrial. Across all patients in the cohort, the median number of prior therapy lines for metastatic disease before CDK4/6i retrial was 3, and 91% of patients in this cohort received CDK4/6i as the immediately preceding therapy before retrial. Of the CDK4/6is, palbociclib (82%) was overwhelmingly used in first-line therapy, while abemaciclib (47%), palbociclib (44%), and ribociclib (9%) were used for retrial.

In Cohort 2 (n=48), all patients progressed through ET + CDK4/6i and underwent CDK4/6i retrial at some point in the future with the same original CDK4/6i but different ET agent. At the time of retrial, 31.2% of patients had bone-only disease, while 10% had brain metastases. Median number of prior treatment lines by time of CDK4/6i retrial was 2. The primary CDK4/6i in this cohort was palbociclib, which was given to 94% of patients for both initial treatment and retrial. 85.4% of patients in this cohort underwent CDK4/6i retrial immediately after progression to the first CDK4/6i regimen.

Cohort 3 (n=127) represented all patients who progressed through ET + CDK4/6i and subsequently underwent CDK4/6i retrial with a different CDK4/6i from their original combination therapy. At the time of retrial, 18% of patients had bone only disease and 11% had brain metastasis. This cohort was overall more heavily pretreated than the other two, as the median number of prior treatment lines by time of retrial was 5, and only 35.4% of patients underwent CDK4/6i retrial as the immediately subsequent therapy line after progression on initial therapy. The overwhelming majority of patients in this cohort were initially treated with palbociclib (96%), with abemaciclib (81.9%) being the primary CDK4/6i of choice for retrial. SERDs (61.5%) were the most common endocrine partner at re-treatment. Separately, 26.5% of patients in this cohort were treated with abemaciclib monotherapy at retrial.

### Time to Treatment Failure with CDK4/6i Retrial

**Figure 2:**
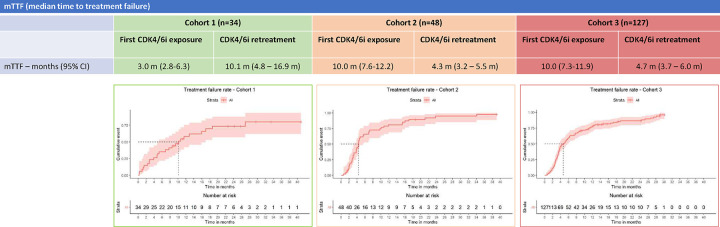
Median Time to Treatment Failure Median TTF for both first CDK4/6i exposure and CDK4/6i retrial are shown in the table above. Below each cohort is the respective survival curves for CDK4/6i retrial. As noted before, median TTF for retrial in Cohort 2 is substantially longer than median TTF for initial exposure. This relationship is inverted for Cohorts 2 and 3, again speaking to the biological difference between Cohort 1 and Cohorts 2 and 3.

**Figure 3:**
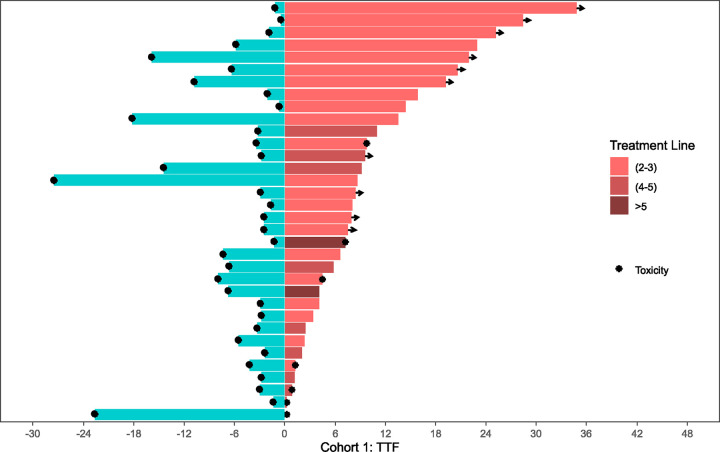
Cohort 1: Time to treatment failure at first CDK4/6i exposure vs. retrial The two-headed swimmer plot for patients in Cohort 1 is shown here. For each patient, both the TTF for initial CDK4/6i exposure (blue, pointing leftward) and for CDK4/6i retrial (pink, pointing rightward) are shown side-by-side. The TTFs for retrial color coded depending upon the treatment line for metastatic disease corresponding to CDK4/6i retrial. TTF2 (2–3) = 2^nd^ or 3^rd^ line; TTF2 (4–5) = 4^th^ or 5^th^ line; TTF2 (>5) = 6^th^ line and beyond.

**Figure 4:**
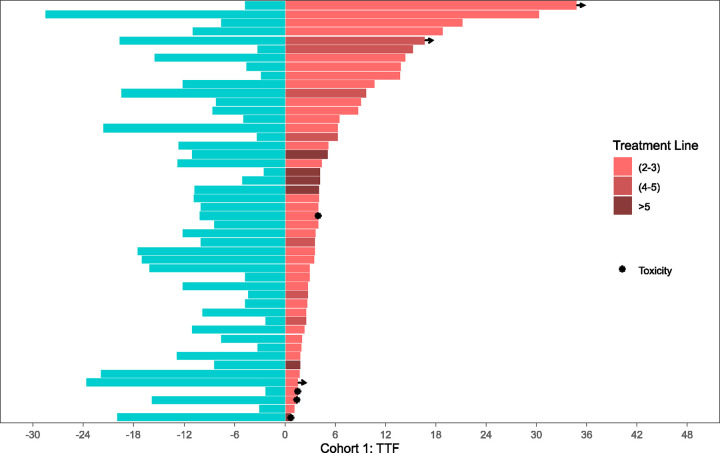
Cohort 2: Time to treatment failure at first CDK4/6i exposure vs. retrial The two-headed swimmer plot for patients in Cohort 2 is shown here, using the same notation as Figure 3. TTF2 (2–3) = 2^nd^ or 3^rd^ line; TTF2 (4–5) = 4^th^ or 5^th^ line; TTF2 (>5) = 6^th^ line and beyond.

**Figure 5:**
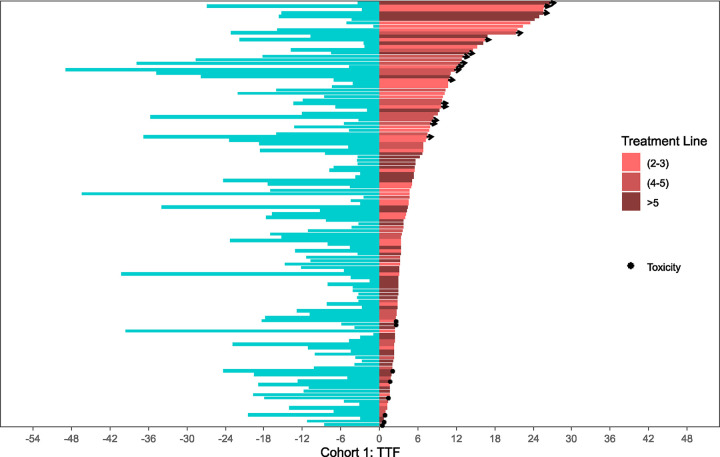
Cohort 3: Time to treatment failure at first CDK4/6i exposure vs. retrial The two-headed swimmer plot for patients in Cohort 3 is shown here, using the same notation as Figure 3 and 4. TTF2 (2–3) = 2^nd^ or 3^rd^ line; TTF2 (4–5) = 4^th^ or 5^th^ line; TTF2 (>5) = 6^th^ line and beyond.

Kaplan-Meier curves summarizing median time to treatment failure (TTF) of both initial CDK4/6i treatment and CDK4/6i retrial are organized per-cohort in Figure 2. Swimmer plots comparing individual TTF for both initial CDK4/6i treatment and CDK4/6i retrial side-by-side per patient are illustrated in Figures 3–5. Median TTF for CDK4/6i retrial in Cohort 1 was 10.1 months (95%CI, 4.8–16.9), in Cohort 2 was 4.3 months (95%CI 3.2–5.5), and in Cohort 3 was 4.7 months (95%CI 3.7–6.0). In Cohorts 2 and 3, most patients stopped treatment with CDK4/6i due to disease progression; otherwise, only 8.3% of patients in Cohort 2 and 6.3% of patients in Cohort 3 stopped due to toxicity. To compare the duration of CDK4/6i retrial to that of initial therapy, we calculated a ratio (which we called the TTF2/TTF1 ratio) by dividing TTF of retrial by TTF of initial CDK4/6i. In Cohort 1, the median TTF2/TTF1 ratio was 1.6, with 60% of patients having a longer TTF on retrial compared to initial treatment (Figure 3). Cohort 2 had a median TTF2/TTF1 ratio of 0.5, and only 29% of patients had a longer TTF2 with CDK4/6i retrial compared to initial treatment (Figure 4). In this cohort, at the time of data censoring, 2 patients (4%) remained on treatment without further progression and 13 patients (27%) had a TTF2 longer than 9 months for CDK4/6i retrial. Cohort 3 had similar numbers to Cohort 2. Cohort 3 had a median TTF2/TTF1 ratio of 0.59, with 32% of patients having a longer CDK4/6i retrial duration than initial treatment (Figure 5). At the time of data censoring, 15 patients in Cohort 3 (11.8%) remained on treatment without further progression and 37 patients (29%) had a TTF2 longer than 9 months on CDK4/6i retrial.

### Best Overall Response

Best overall response (BOR) to first exposure and retrial of CDK4/6i are summarized in Table 2. In Cohort 1, where patients had not demonstrated progression on a CDK4/6i yet, 29% of the patients had radiographic response, 29% had stable disease (SD), 15% had progression of disease (PD), and 26% were non-evaluable (their treatment changed before first re-staging scan) in response to CDK4/6i retrial. In Cohort 2, where all patients had progressed on a preceding line of CDK4/6i, 15% had radiographic response, 25% had SD, and 48% had PD by time of first restaging scans for CDK4/6i retrial, while 12% of patients were non-evaluable. In Cohort 3, again where all patients had previously progressed on a prior line involving CDK4/6i, 22% of patients had radiographic response, 24% had SD, and 41% had PD by time of first restaging scans for CDK4/6i retrial, and 13% of patients were non-evaluable. All patients who had radiographic response were initially treated with palbociclib, and 82% of these patients were switched to abemaciclib for CDK4/6i retrial, including 6 patients (21% of responders) who were treated with abemaciclib monotherapy.

### Univariate and Multivariate Analysis

We conducted Cox regression for survival analysis to both compare TT1 to TTF2 and to determine variables associated with a higher TTF2. In Cohort 1, initial CDK4 exposure (TTF1) was significantly shorter than CDK4/6i retrial (TTF2) (HR 0.40, 95%CI 0.24–0.70, p=0.001); in Cohort 2, TTF1 was not significantly different from TTF2 (HR 1.41, 95%CI 0.94–2.14, p=0.09); in Cohort 3, TTF1 was significantly longer than TTF2 (HR 1.44, 95%CI 1.11–1.87, p=0.007). For Cohort 2, none of the variables tested on univariate or multivariate Cox regression were significantly associated with a higher TTF2 (variables included: presence of bone-only disease, presence of brain metastases at treatment, treatment line of CDK4/6i retrial, TTF of initial CDK4/6i treatment, and best response to initial CDK4/6i treatment by PRISSMM criteria). For Cohort 3, using the same variables, univariate Cox regression found having bone-only metastases to be significantly associated with higher TTF2 (HR 0.57, 95%CI 0.31–0.83, p=0.03), while having brain metastases was associated with significantly lower TTF2 (HR 1.78, 95%CI 1.49–2.07, p=0.048). However, on multivariate Cox regression, these variables lost statistical significance, yielding no variables associated with higher TTF2 similar to Cohort 2; however, having bone-only disease trended towards significance for a higher TTF2 (HR0.60, 95%CI 0.35–1.02, p=0.06).

### Somatic Tumor Mutation Profiling and Associations with Retrial Benefit

In an exploratory analysis, we compared the somatic tumor mutation profiles (based on hybrid-capture panel-based NGS using MSK-IMPACT)^13^ of patients in Cohort 3 that had a TTF2 at CDK4/6i retrial shorter than 4 months (representing clinically resistant disease on par with the median PFS of the placebo arm in the PALOMA-3 trial) to those with a TTF2 at the time of retrial longer than 9 months (representing clinically responsive disease similar to the median PFS of the treatment arm in the PALOMA-3 trial).^14^ From the 53 patients with a TTF2 <4 months and the 34 patients with a TTF2 >9 months, we were able to identity 50 patients where somatic mutation profiling was done prior to any CDK4/6i exposure, 22 patients with profiling done in between the two CDK4/6i regimens, and 15 patients with genomic data collected post-progression to CDK4/6i retrial.

**Figure 6 –:**
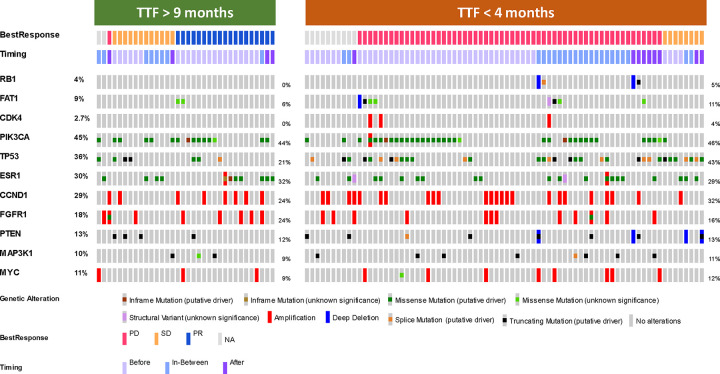
Genomic Alterations in patients with short and long TTF to CDK4/6i retrial in Cohort 3 Somatic tumor mutation profiles of patients in Cohort 3 that had good response (>9 months TTF) and poor response (<4 months TTF) for CDK4/6i retrial. Each column represents an individual patient, organized first by BOR by PRISSMM criteria then by timing of mutational profile sample (Before first CDK4/6i, In-Between initial exposure and retrial, or After CDK4/6i retrial). *RB1* and *FAT1* loss of function mutations as well as *CDK4* amplifcations were seen exclusively in patients with TTF<4months. Two patients in the TTF>9 months had *FAT1* mutations that were variants of unknown significance. Other classical ER+ MBC resistance mutations, such as those in *TP53, PIK3CA*, and *ESR1* were fairly evenly distributed between the two subgroups.

The genomic results for these 87 patients are presented in the Oncoprint shown in Figure 6. As expected, patients with a shorter TTF2 to CDK4/6i retreatment had a higher frequency of genomic changes previously described as potential resistance mechanisms to CDK4/6i, such as *TP53* mutations (43% in low TTF2 cohort vs 21% in high TTF2 cohort), *CDK4* amplifications (4% vs 0%), *RB1* loss, (5% vs 0%) and *FAT1* loss-of-function mutations (5% vs 0%).^15^ Notably, all patients with *RB1* mutations acquired them after initial CDK4/6i exposure and all presented with immediate PD with CDK4/6i retrial. None of the patients with prolonged TTF2 to CDK4/6i re-treatment had loss-of-function mutations in *RB1* or *FAT1,* although two patients in this group did develop *FAT1* variants of unknown significance (VUS) after initial CDK4/6i exposure. Both groups of patients with TTF2 <4 months and >9 months had near equal prevalence of mutations commonly seen after combination ET + CDK4/6i therapy, such as *PIK3CA* and *ESR1* mutations.

## DISCUSSION

In this single-center retrospective cohort study, we report our experience with CDK4/6i retrial for the treatment of heavily pre-treated ER+ MBC. Within our center, we identified three discrete cohorts to describe CDK4/6i retrial as a treatment strategy for this disease, and we report the real-world implications of this treatment strategy despite prior exposure to and treatment failure of CDK4/6i based regimens.

Of the cohorts identified, it is unsurprising that Cohort 1, which contained patients who had to stop initial CDK4/6i due to toxicity as opposed to poor efficacy, represents separate biology when compared to patients who had to stop initial CDK4/6i due to disease progression on therapy. The patients in Cohort 1 experienced both comparably higher rates of clinical response and treatment-limiting toxicity compared to patients that had disease progression on initial CDK4/6i exposure (20.6% discontinuation rate due to toxicity in Cohort 1 compared to 8.3% in Cohort 2 and 6.3% in Cohort 3). Overall, this suggests that CDK4/6i retrial after initial treatment failure due to toxicity is viable and should be considered as a further line of therapy in this patient population, with the caveat that the risk of similar toxicity is nontrivial. Most of this cohort switched CDK4/6i, but some patients underwent retrial with palbociclib again, though at a lower starting dose and in a later treatment line. Of the 7 patients in this cohort that had to stop CDK4/6i retrial due to treatment toxicity, 6 patients (85.7%) still had to stop retrial therapy due to the same toxicity that prompted discontinuation of their initial CDK4/6i even though 5 (71.4%) patients switched to a different CDK4/6i. These results suggest that there exists a subset of patients that are uniquely sensitive to toxicity from CDK4/6i’s as a class, and that switching individual agents may still not be enough to abrogate this toxicity.

Regarding efficacy of a CDK4/6i retrial strategy post-progression, the three prospective trials mentioned above (PACE, PALMIRA, and MAINTAIN), have altogether still not provided conclusive evidence whether CDK4/6 inhibition adds any differential efficacy compared to next line endocrine therapy alone, mainly due to conflicting results between the trials in question. In all three trials, most patients had previous exposure and progression on palbociclib, which is one argument to as why MAINTAIN, which changed both the endocrine therapy partner and the CDK4/6i in subsequent treatment lines, yielded a positive result. Further, while the three main CDK4/6i approved for ER+ MBC were initially considered equivalent based upon the comparable PFS data from the initial trials, longer-term follow-up showed differential OS benefit between the three agents, with abemaciclib and ribociclib showing comparable median OS’s of 67.1 months in MONARCH 3^16^ and 63.9 months in MONALEESA-2,^17^ respectively, but palbociclib showing a notably shorter OS of 53.9 months in PALOMA-2.^18^ As a result, since direct head-to-head data does not exist and it is not known why there is an OS difference despite similar PFS, there is growing suspicion that the different CDK4/6is are not equivalent, with multiomic studies demonstrating key molecular differences and resistance patterns between the three agents.^19^ These altogether raise the additional question of whether switching CDK4/6i’s upon retreatment provides additional clinical value.

Due to the retrospective nature of our study and lack of a comparator arm, our data unfortunately cannot clarify this question further, but it does help frame the trial results through a real-world lens and may add more context for the disparate trial results. Given that a small minority of patients in both Cohort 2 and 3 discontinued treatment due to toxicity, the median TTF2s for both Cohorts 2 (4.3 months) and 3 (4.7 months) roughly approximate PFS, which in turn also approximates in scale the median PFS’s seen in these trials: PACE 4.6 months,^10^ PALMIRA 4.2 months,^11^ MAINTAIN 5.2 months.^12^ The specific question of whether changing CDK4/6i on retrial yields differential efficacy is of particular clinical interest; a separate multicenter retrospective analysis investigating 87 patients specifically treated with abemaciclib after progression on either palbociclib or ribociclib similarly showed a median PFS of 5.3 months for these patients and also suggested that abemaciclib remains a viable treatment strategy for CDK4/6i retrial.^20^ Our data from Cohort 3 corroborates these findings with a larger sample size, but both studies lacked direct comparator arms (our study includes Cohort 2 as the subgroup of patients who did not switch CDK4/6i, but our analysis was not powered for direct comparison of Cohort 2 and 3, and there were a number of clinical differences that may confound any PFS differences, notably that Cohort 3 was on the whole more heavily pre-treated but also had a greater proportion of patients with TTF2 >9 months). Nevertheless, a number of randomized phase III trials are underway that are prospectively investigating abemaciclib after progression on a prior CDK4/6i with a number of different endocrine therapy partners, namely postMONARCH,^21^ EMBER-3,^22^ and ELAINE 3.^23^ The results of these trials will hopefully provide more definitive data to guide clinical practice.

Our data does instead clearly demonstrate that this patient population is heterogenous, and the clinical and genomic complexity of this group warrants patient assessment on an individualized basis regarding the appropriateness of CDK4/6i retrial as a treatment strategy. Specifically, there was a sizable proportion of patients that derived significant benefit (TTF2>9 months) in both Cohort 2 (27.1%) and Cohort 3 (29.7%). While not significantly associated with longer TTF2 on multivariate analysis, both the presence of bone-only disease and the lack of brain metastases were significantly associated with longer response on univariate analysis and are both otherwise conventionally known to portend overall better outcomes. Genomically, *TP53* mutations were over-represented among patients with low TTF2, and well-known CDK4/6i resistance mutations such as *CDK4* amplification, *RB1* loss, and *FAT1* loss of function^24^ were seen exclusively in patients with low TTF2. Due to the overall low number of cases, this was a descriptive analysis that could be validated in future randomized studies but does suggest that the presence of known resistance mutations to ET + CDK4/6i after initial therapy would predict poor response to a CDK4/6i retrial, regardless of whether the same or a different CDK4/6i is used. Taken together, these clinical and genomic characteristics may be useful metrics in selecting patients more likely to benefit from CDK4/6i retrial while also identifying those that would likely have poor response.

Our study has a number of limitations. Most notably, the retrospective nature limits our ability to make definitive conclusions, as does our lack of an endocrine therapy only comparator arm. However, despite this, our results from Cohort 3, where the CDK4/6i was changed but ET was not for most cases, suggest that CDK4/6 inhibition is biologically relevant to the treatment results and the effects seen are not simply from ET alone. This is further supported by our genomic results, which show differential enrichment of classical CDK4/6i resistance mutations in the subgroup of patients with lower TTF2 alongside relative parity of *ESR1* mutations in both the higher TTF2 and lower TTF2 subgroups; if treatment effect was driven primarily by ET, we would expect this mutation distribution to be reversed. Another limit of our study is also the age and breadth of the data collection period. While the broad data analysis period is an independent strength because it allows assessment of longer-term follow-up for a larger number of patients, it is also a weakness given the rapid pace at which standard of care changes and new options become available. A manifestation of this is the fact that the overwhelming majority of our patients were treated with palbociclib as first CDK4/6i since it was what was available at the time, and providers did not have the newer OS data of various CDK4/6i to help guide agent selection. Another aspect of the data’s age that may affect overall generalizability is that our study cohort therefore disproportionately selected for patients with long-standing ER+ MBC who were being treated in a time where the main treatment options were still successive lines of cytotoxic chemotherapies, and newer targeted agents (such as antibody-drug conjugates or newer kinase inhibitors) were not available.

In summary, this single center, retrospective study presents proof of feasibility and tolerability of CDK4/6i retrial in a large cohort of patients with heavily pre-treated ER+ MBC. In line with prior published data, our data suggests that a subset of patients might benefit from CDK4/6i retrial and that using a different CDK4/6i at time of retrial may be beneficial. First, for patients who stopped a CDK4/6i due to toxicity, rotation to a different CDK4/6i or rechallenge with the same CDK4/6i in a later treatment line is both a viable and effective strategy, with favorable TTF and toxicity profiles for the majority of patients on CDK4/6i retrial. For patients who have progression on a CDK4/6i, individualized assessment at both the clinical and molecular levels is necessary for selection of patients most likely to derive benefit from a retrial strategy. Our data is concordant with conventional knowledge that patients with bone-only disease tend to benefit from CDK4/6i retrial more compared to those that have visceral metastases, even though it only trended towards statistical significance in this respect. Alternatively, *TP53* mutations, *CDK4* amplifications, and *RB1* or *FAT1* loss of function mutations may be molecular biomarkers predictive of CDK4/6i retrial failure. Further investigation of the clinical and genomic features of response and resistance to CDK4/6 inhibition is necessary to answer many of the remaining questions about this treatment strategy. Overall, several phase 3 trials are currently underway to answer these many questions, and we eagerly await their results to more definitively address them.

## METHODS

### Patients

Eligible patients were 18 years of age or older, had biopsy-confirmed unresectable stage III or stage IV ER+ breast cancer, were treated at our institution, and received two or more lines of treatment for advanced disease, with at least two prior lines containing a CDK4/6i. Patients with initial ER+/HER2+ breast cancer were excluded unless their disease reverted to a HER2 negative state by the time of CDK4/6i exposure.

### Study Design

After obtaining a waiver of consent from the institutional review board, we performed a single-center, retrospective analysis of patients treated between May 2014 to December 2020 with at least two separate treatment lines containing a CDK4/6i for advanced ER+ breast cancer. Patients were identified through the MSK Breast Cancer Translational Platform (MSK-BCTP)^7^ and the MSK pharmacy system. Detailed review of electronic medical records (EMR) was done by two independent physicians. Efficacy outcomes such as BOR and TTF were extrapolated from the EMR. For each line of treatment in a patient’s case: start date, end of treatment date, and reason for therapy discontinuation (toxicity, progression, death or other) were annotated, standardized, and stored in our REDCap (Research Electronic Data Capture) platform. Somatic tumor mutation profiling via targeted hybrid-capture based NGS (MSK-IMPACT)^13^ was recorded for pre-treatment (before any CDK4/6i exposure), inter-treatment (after only one treatment line containing CDK4/6i), and post-treatment (after all treatment lines containing CDK4/6i) biopsies when available.

Efficacy outcomes were evaluated in 3 different patient cohorts. For the number of heavily pre-treated patients that had been exposed to CDK4/6i in 3 or more treatment lines by time of data analysis, we extracted data from their two most recent lines containing CDK4/6i, with the earlier line counting as their “initial” treatment and the later line counting as “retrial” for the purposes of our analysis. We first divided all patients based upon whether their initial CDK4/6i-containing line of therapy was discontinued due to treatment toxicity or progression of disease (POD (Figure 1). Cohort 1 therefore represents all patients who had incomplete exposure to CDK4/6i therapy at some point due to toxicity but subsequently were treated with either the same or separate CDK4/6i in a later treatment line. Among the patients who had stopped initial CDK4/6i therapy due to POD, these patients were further divided based upon whether their subsequent treatment with CDK4/6i included the same or a different CDK4/6i. Cohort 2 therefore represents all patients with POD on initial CDK4/6i who were subsequently re-treated with the same CDK4/6i but now combined with a separate endocrine therapy partner. Cohort 3 represents all patients with POD on initial CDK4/6i who were instead treated with a different CDK4/6i with the same or different endocrine partner in a later line of treatment.

### Outcomes

The primary objective of this study was to evaluate TTF on CDK4/6i re-treatment in the 3 different pre specified cohorts. TTF was defined as the time in months from when a patient started CDK4/6i retreatment to discontinuation of CDK4/6i for any reason, including disease progression, treatment toxicity, or death. We did not choose PFS as our endpoint because PFS would not adequately characterize the potential toxicity of this treatment strategy, which is something directly relevant to clinical practice. As a secondary end point, we evaluated tumor response to CDK4/6i retreatment in each of the 3 cohorts. Tumor response was assessed based on clinician assessment of response and investigator imaging review, as per PRISSMM criteria. Patients that stopped CDK4/6i treatment before a re-staging image or only had non-measurable lesions were classified as non-evaluable patients.

To better understand potential associations between certain clinical variables and response to CDK4/6i retrial, we included the following variables in our analysis: presence of bone only disease, presence of brain metastasis, number of disease sites, treatment line of CDK4/6i retrial, time to progression on initial CDK4/6i treatment, and best response to initial CDK4/6i treatment by PRISSMM criteria.^25^ As part of exploratory analysis, we also conducted a detailed genomic description of patients with the most disparate clinical outcomes and compared the genomic profiles of those with short (less than 4 months) to prolonged (more than 9 months) TTF to assess for any potential trends. These time points were chosen as a rough comparison to the results of the PALOMA-3 trial, which investigated palbociclib + fulvestrant vs. placebo + fulvestrant in patients with MBC and reported PFSs of 9.5 months in the treatment arm vs. 4.6 in the placebo arm.^14^

### Statistical Analysis

TTF was estimated using Kaplan-Meier methods, and survival curves were compared using long-rank test. The association of risk factors with TTF was analyzed using Cox proportional hazards method. Associations between clinical variables and outcomes were assessed with both univariate (using non-parametric paired statistical tests) and multivariate (using logistic regression) analyses. All statistical analysis was performed using R Statistical Software.

## Figures and Tables

**Table 1:** Baseline Patient Characteristics:

Patient Characteristics						

	Cohort 1 (n 34)		Cohort 2 (n 48)		Cohort 3 (n 127)	

	First CDK4/6i exposure	CDK4/6i re-treatment	First CDK4/6i exposure	CDK4/6i re-treatment	First CDK4/6i exposure	CDK4/6i re-treatment

***Prognostic Markers***						

Median Age	**61**	**62**	**56**	**58**	**61**	**63**

Bone Only Metastases*	**9 (26%)**	**9 (26%)**	**18 (37.5%)**	**15 (31.2%)**	**40 (31.2%)**	**23 (18%)**

Sites of metastatic disease						
1	**18 (53%)**	**18 (53%)**	**22 (46%)**	**17 (35%)**	**49 (39.6%)**	**25 (19.7%)**
2	**6 (18%)**	**4 (12%)**	**11 (23%)**	**9 (19%)**	**35 (27.6%)**	**31 (24.4%)**
3	**6 (18%)**	**8 (24%)**	**10 (21%)**	**12 (25%)**	**20 (15.8%)**	**29 (22.9%)**
=4	**4 (12%)**	**4 (12%)**	**5 (10%)**	**10 (21%)**	**23 (18.1%)**	**42 (33.1%)**

Brain Metastasis	**1 (3%)**	**1 (3%)**	**3 (6%)**	**5 (10%)**	**10 (7.9%)**	**14 (11%)**

***Endocrine Partner***						

Aromatase Inhibitor	**22 (65%)**	**16 (47%)**	**44 (92%)**	**1 (2%)**	**68 (54%)**	**14 (11%)**

SERD	**11 (32%)**	**15 (44%)**	**1 (2%)**	**45 (94%)**	**51 (40%)**	**78 (61%)**

Tamoxifen	**0**	**0**	**0**	**1 (2%)**	**0**	**1 (0.7%)**

No Endocrine partner	**1 (3%)**	**3 (9%)**	**3 (6%)**	**1 (2%)** ^μ^	**8 (6%)** ^ß^	**34 (27%)**

***CDK4/6i***						

Palbociclib	**28 (82%)**	**15 (44%)**	**45 (94%)**	**45 (94%)**	**122 (96.1%)**	**4 (3.2%)**

Abemaciclib	**4 (12%)**	**16 (47%)**	**3 (6%)**	**3 (6%)**	**4 (3.2%)**	**104 (81.9%)**

Ribociclib	**2 (6%)**	**3 (9%)**	**0**	**0**	**1 (0.8%)**	**19 (15%)**

***Treatment Sequencing***						

CDK4/6i retrial immediately after initial CDK4/6i failure	**31 (91%)**		**41 (85.4%)**		**45 (35.4%)**	

Median Lines of Therapy for Metastatic Disease	**1**	**3**	**1**	**2**	**2**	**5**

First line	**19 (56%)**	**0**	**29 (60%)**	**0**	**43 (33.9%)**	**0**

Second line	**6 (18%)**	**16 (47%)**	**7 (15%)**	**28 (58%)**	**28 (22.1%)**	**17 (13.4%)**

Third line	**1 (3%)**	**7 (21%)**	**4 (8%)**	**6 (13%)**	**18 (14.2%)**	**23 (18.1%)**

Fourth and beyond	**8 (24%)**	**11 (32%)**	**8 (17%)**	**14 (29%)**	**38 (30%)**	**87 (68.5%)**

* Patient with bone lesions and breast primary lesion and/or lymph node involvement were included as bone only as far as no presence of visceral disease.

μ One patient received bicalutamide as endocrine partner

? Six patients received bicalutamide as endocrine partner

**Table 2:** Best Overall Response by Cohort

Best Overall Response (PRISSMM Criteria)						

	Cohort 1 (n 34)		Cohort 2 (n 48)		Cohort 3 (n 127)	

	First CDK4/6i exposure	CDK4/6i retreatment	First CDK4/6i exposure	CDK4/6i retreatment	First CDK4/6i exposure	CDK4/6i retreatment

**Disease Progression**	0	5 (15%)	14 (29.1%)	23 (47.9%)	39 (30.7%)	52 (40.9%)

**Stable Disease**	10 (29%)	10 (29%)	6 (12.5%)	12 (25%)	30 (23.6%)	31 (24.4%)

**Radiological Benefit**	11 (32%)	10 (29%)	25 (52.0%)	7 (14.5%)	54 (42.5%)	28 (22.0%)

**Non-evaluable (Treatment changed before first re-staging image)**	13 (38%)	9 (26%)	3 (6.2%)	6 (12.5%)	4 (3.1%)	16 (12.6%)

Best overall response by radiographic PRISSMM criteria is outlined by cohort in the table above. Patients that were nonevaluable were mainly patients that did not get radiographic imaging to determine disease state prior to changing therapies.

## Data Availability

Data are available upon reasonable request at the discretion of the corresponding authors. Access to datasets used in this study should be requested directly from the corresponding authors and will involve data access request forms via Memorial Sloan Kettering Cancer Center. Subject to the institutional review boards’ ethical approval, unidentified data may be made available as a test subset. Data analysis methods have been described thoroughly in the Methods section so they can be independently replicated.
